# Down syndrome, paternal age and education: comparison of California and the Czech Republic

**DOI:** 10.1186/1471-2458-5-69

**Published:** 2005-06-17

**Authors:** Dagmara Dzurova, Hynek Pikhart

**Affiliations:** 1 Faculty of Science, Charles University, Czech Republic; 2 School of Public Health, University of California, Berkeley, USA; 3 Department of Epidemiology and Public Health, University College London, UK

## Abstract

**Background:**

The association between maternal age and risk of Down syndrome has been repeatedly shown in various populations. However, the effect of paternal age and education of parents has not been frequently studied. Comparative studies on Down syndrome are also rare. This study evaluates the epidemiological characteristics of Down syndrome in two culturally and socially contrasting population settings, in California and the Czech Republic.

**Methods:**

The observed live birth prevalence of Down syndrome was studied among all newborns in the California counties monitored by California Birth Defects Monitoring Program from 1996 to 1997, and in the whole Czech Republic from 1994 to 1998. Logistic regression was used to analyze the data.

**Results:**

A total of 516,745 (California) and 475,834 (the Czech Republic) infants were included in the analysis. Among them, 593 and 251, respectively, had Down syndrome. The mean maternal age of children with Down syndrome was 32.1 years in California and 26.9 years in the Czech Republic. Children born to older mothers were at greater risk of Down syndrome in both populations. The association with paternal age was mostly explained by adjusting for maternal age, but remained significant in the Czech Republic. The association between maternal education and Down syndrome was much stronger in California than in the Czech Republic but parental age influences higher occurrence of Down syndrome both in California and in the Czech Republic.

**Conclusion:**

The educational gradient in California might reflect selective impact of prenatal diagnosis, elective termination, and acceptance of prenatal diagnostic measures in Californian population.

## Background

Down syndrome (also called trisomy 21 or trisomy of chromosome 21), is the most common chromosome abnormality in newborns. The disease is associated with mental retardation, immune system disorders, autoimmune problems, premature aging and Alzheimer disease at the age of 30–40 years [1,2]. The association between maternal age and risk of having a Down syndrome pregnancy was first published in 1933 [3]. In 1959 the presence of an extra chromosome 21 was identified [4]. In 1966, the first chromosome analysis of amniotic fluid cells was published [5]. The first report of the antenatal diagnosis of Down syndrome was published two years later [6].

Many studies have been conducted to increase understanding of the Down syndrome epidemiology and its geographic variations [7-11]. The relation of advanced maternal age to an increased risk of Down syndrome has been established, but the effects of other risk factors have not been confirmed [12,13]. Given the current level of knowledge, neither the conception of children with Down syndrome nor their birth can be prevented but several screening programs do exist.

Although Down syndrome affects a relatively small number of families directly, internationally it is discussed with millions of parents every year when they are offered prenatal screening. This is often the first time the individuals are confronted with questions about the usefulness and value of genetic testing [14,15]. The diagnosis of Down syndrome is made by chromosomal analysis, which can be initiated prenatally (in the first or second trimester of pregnancy) due to given risk factors for pregnancy, or postnatally due to the characteristic appearance of the newborn child.

Because routine prenatal screening is based on the assumption that it is reasonable for prospective parents to choose to prevent a life with Down syndrome, the proportion of people with Down syndrome in the future will be based both on the development of prenatal screening and on the personal choices of prospective parents [14-17].

In this study, we analyze the epidemiological characteristics of Down syndrome in two culturally and socially contrasting settings, in California and the Czech Republic. The Californian population represents an advanced democratic society with a long tradition of positive attitudes towards people with disabilities [18]. The second is a post-communist society in transition, the Czech population. In the Czech Republic, children with Down syndrome were often referred at birth to residential institutions before year 1989 (the "Velvet revolution"). The communist party propaganda promoted better health through removing people with disabilities from mainstream society. During the past 15 years of societal transformation, negative attitudes have changed. Most of the positive improvements, social acceptance, and the quality of life of people with disabilities have been the results of parental support. As a result of parental support and activity, children with Down syndrome are gradually integrated to the general population. The same trend was recognized in the United States, but it was 30–40 years earlier [19].

The objective of this study is to quantify the effects of different demographic factors on the prevalence of live births with Down syndrome, to examine possible interactions between them, and to compare effects of these factors in two different populations. To do this, we used data from a population-based registry in California, and from birth and congenital anomalies registers in the Czech Republic. Our main focus was on parental age and education. Although the effect of maternal age as a risk factor for Down syndrome is well known, the role of paternal age and maternal education has not been clearly established. Additionally, there have not been many epidemiological studies assessing the association between Down syndrome and parental demographic factors in Central and Eastern Europe.

## Methods

### Samples

Individual anonymous records of births and congenital anomalies were collected from two populations.

The Californian sample comes from the California Birth Defects Monitoring Program data (CBDMP), representing all births from January 1, 1996, to December 31, 1997 in hospitals in the California counties monitored by the registry (n = 516,745). In addition to birth data, the CBDMP, a regional population-based registry of congenital anomalies, recorded information about birth defects within the first year of life for all newborns registered by this program.

The Czech sample includes all live births reported to the Czech Statistical Office between January 1, 1994 and December 31, 1998 (n = 475,834). For the purpose of this study, birth registry data were linked to the Czech Congenital Anomalies Register by national personal identity numbers. The mandatory statistical records are kept for all children with congenital anomalies up to 15 years of age. The birth/congenital anomalies data set was linked at the Institute of Health Informatics and Statistics of the Czech Ministry of Health (for research project No. 403/00/1521). The linkage was successful for 95% of Down syndrome cases.

For our analysis, we used all live births with gestational age 25 weeks or longer (25 weeks was the lowest recorded gestational age in the Czech sample). Therefore, 2,366 Californian babies with recorded gestational age lower than 25 weeks (including 4 Down syndrome cases) were excluded from the analysis.

### Variables

We used only variables available in both datasets. For this reason we did not use, for example, ethnicity because this variable is not available in the Czech register. Some variables were categorized for the purpose of the analysis.

Maternal and paternal age at child birth was used as continuous or categorical variable. When used as categorical, the age was classified into ten 3-year age groups (19 years or below; 20–22, 23–25, 26–28, 29–31, 32–34, 35–37, 38–40, 41–43, and over 44 years). We used 10 categories because we wanted to assess possible non-linear trends between parental age and prevalence of Down syndrome, and our large samples allowed the use of such number of categories. Maternal education at the birth of child was classified into four categories: primary, vocational, secondary and university.

### Analysis

All live-born cases of Down syndrome (diagnosed either pre- or postnatally) were used as the outcome in our analysis. The effect of parental characteristics on occurrence of Down syndrome was quantified by logistic regression. First, crude odds ratios were calculated for each of the independent variables available in both datasets (maternal and paternal age, education of mother, and sex of infants). Then, all characteristics, except paternal age, were entered into one model to assess their independent effects, and adjusted odds ratios were calculated. Finally, we included paternal age in the model, and fully adjusted odds ratios were estimated.

All analyses were carried out using the SPSS (SPSS Inc, Chicago, USA) and STATA (Stata Corp, College Station, Texas, USA) statistical packages. The use of the data was in accordance with the statutory obligations to protect confidentiality. Individuals could not be identified from the data provided for analysis.

## Results

The crude observed live birth prevalence of Down syndrome in California was 11.5 per 10,000 births in 1996–97 (Table 1). This figure represents 1 case of Down syndrome in every 870 live births. In the Czech Republic, the live birth prevalence of Down syndrome was 5.3 per 10,000 live births in 1994–98 (Table 1). This figure represents 1 case of Down syndrome in every 1,900 live births. There was a slightly higher proportion of boys with Down syndrome in California (12.4 boys vs. 10.5 girls per 10,000 live births) and in the Czech Republic (5.9 boys vs. 4.6 girls per 10,000 live births).

**Table 1:** Numbers of newborns, children with Down syndrome, and prevalence rates of Down syndrome, California (1996–97) and the Czech Republic (1994–98)

	**California**					**Czech Republic**				
	
	All children		Children with DS			All children		Children with DS		
	
	No.	% of births	No.	% of births	Prevalence per 10 000	No.	% of births	No.	% of births	Prevalence per 10 000
	
*Sex of infants*										
Boy	264 321	51.2	328	55.3	12.4	244 503	51.4	144	57.4	5.9
Girl	252 408	48.8	265	44.7	10.5	231 331	48.6	107	42.6	4.6
Unknown	16		0			0		0		

*Education of mother*										
Primary	85 615	16.6	157	26.5	18.3	64 774	13.6	34	13.5	5.2
Vocational	107 633	20.8	96	16.2	8.9	199 260	41.9	110	43.8	5.5
Secondary	234 312	45.3	251	42.3	10.7	168 659	35.4	80	31.9	4.7
University	83 681	16.2	77	13.0	9.2	43 135	9.1	27	10.8	6.3

Unknown	5 504		12			6		0		

*Maternal age*										
-19	65 058	12.6	31	5.2	4.8	46 236	9.7	17	6.8	3.7
20–22	73 102	14.1	46	7.8	6.3	118 195	24.8	57	22.7	4.8
23–25	79 444	15.4	55	9.3	6.9	121 827	25.6	50	19.9	4.1
26–28	83 791	16.2	56	9.4	6.7	85 173	17.9	40	15.9	4.7
29–31	78 435	15.2	64	10.8	8.2	52 998	11.1	28	11.2	5.3
32–34	64 442	12.5	101	17.0	15.7	28 689	6.0	27	10.8	9.4
35–37	42 301	8.2	75	12.6	17.7	14 171	3.0	14	5.6	9.9
38–40	21 289	4.1	83	14.0	39.0	6 431	1.4	7	2.8	10.9
41–43	7 173	1.4	68	11.5	94.8	1 818	0.4	6	2.4	33.0
44+	1 527	0.3	14	2.4	91.7	296	0.1	5	2.0	168.9

Unknown	183		0			0		0		

*Paternal age*										
-19	24 635	4.8	14	2.4	5.7	6 670	1.4	3	1.2	4.5
20–22	47 498	9.2	30	5.1	6.3	49 493	10.4	14	5.6	2.8
23–25	64 126	12.4	41	6.9	6.4	87 675	18.4	43	17.1	4.9
26–28	74 838	14.5	54	9.1	7.2	88 677	18.6	41	16.3	4.6
29–31	75 282	14.6	82	13.8	10.9	68 831	14.5	29	11.6	4.2
32–34	69 024	13.4	76	12.8	11.0	42 989	9.0	24	9.6	5.6
35–37	51 791	10.0	75	12.6	14.5	23 953	5.0	21	8.4	8.8
38–40	32 849	6.4	46	7.8	14.0	14 105	3.0	14	5.6	9.9
41–43	18 406	3.6	66	11.1	35.9	7 402	1.6	7	2.8	9.5
44+	18 286	3.5	66	11.1	36.1	6 610	1.4	9	3.6	13.6

Unknown	40 010		43			79 429		46		

***Total***	**516 745**	**100**	**593**	**100**	**11.5**	**475 834**	**100**	**251**	**100**	**5.3**

In California, mean maternal age (SD) was 27.2 (6.5) years for mothers of all children and 32.1 (7.3) years for mothers of children with Down syndrome, whereas in the Czech Republic, it was 25.1 (4.9) and 26.9 (6.3) years. The maternal age difference between non-Down syndrome and Down syndrome children was significant in both Californian and Czech samples (t = 18.84, p < 0.001 for California, and t = 6.11, p < 0.001 for the Czech Republic). In California, the highest proportion of all children was born to mothers aged 23–31, and of children with Down syndrome to mothers aged 32–40. The live birth prevalence rate of Down syndrome was higher among older mothers (Table 1). In the Czech Republic the largest proportion of newborns and newborns with Down syndrome was among mothers aged 20–25 years. Prevalence rates of Down syndrome substantially increased with increasing maternal age (Table 2). There is a large difference in proportion of Down syndrome babies born to younger women (< 35 years); in California it was 59.5% and in the Czech Republic 87.3% of Down syndrome babies.

**Table 2:** Odds ratios (95% confidence interval) of occurrence of Down syndrome California, 1996–97

	Crude	95% CI		Fully adjusted*	95% CI	
		Lower	Upper		Lower	Upper
*Sex of infants*						
Boy	1			1		
Girl	0.85	0.72	1.00	0.84	0.71	0.99

*Education of mother*						
Primary	1.99	1.52	2.62	2.80	2.12	3.72
Vocational	0.97	0.72	1.31	2.24	1.62	3.10
Secondary	1.16	0.90	1.50	1.87	1.44	2.44
University	1			1		

P for trend	*<0.001*			*<0.001*		

*Maternal age*						
-19	1			1		
20–22	1.32	0.84	2.08	1.66	0.95	2.92
23–25	1.45	0.94	2.26	2.05	1.14	3.68
26–28	1.40	0.90	2.17	2.18	1.20	3.99
29–31	1.71	1.12	2.63	2.74	1.49	5.04
32–34	3.29	2.20	4.92	5.47	3.00	9.98
35–37	3.72	2.45	5.66	6.27	3.37	11.64
38–40	8.21	5.43	12.40	12.34	6.61	23.05
41–43	20.07	13.12	30.71	28.42	14.98	53.91
44+	19.40	10.30	36.55	27.57	12.22	62.21
*Paternal age*						
-19	1			1		
20–22	1.11	0.59	2.10	0.85	0.44	1.67
23–25	1.13	0.61	2.06	0.69	0.35	1.37
26–28	1.27	0.71	2.29	0.65	0.32	1.31
29–31	1.92	1.09	3.38	0.83	0.41	1.66
32–34	1.94	1.10	3.43	0.67	0.33	1.37
35–37	2.55	1.44	4.51	0.68	0.33	1.40
38–40	2.47	1.36	4.49	0.54	0.26	1.13
41–43	6.33	3.55	11.27	1.02	0.49	2.11
44+	6.37	3.58	11.34	0.86	0.41	1.79

Note: * -Adjusted for all variables in the Table.

In California, mean age (SD) of fathers of all children and children with Down syndrome was 35.4 (19.7) and 38.7 (18.6) years, whereas in the Czech Republic it was 28.2 (5.7) and 30.2 (6.9). The difference in paternal age between non-Down syndrome and Down syndrome babies was highly significant in both samples: t = 12.94 (P < 0.001) in California and t = 5.04 (P < 0.001) in the Czech Republic. In California, the highest percentage of all newborns was among fathers 26–34 years old, while the highest percentage of children with Down syndrome among fathers 29–37 years old. In the Czech Republic it was among 23–31 year olds for both all newborn babies and babies with Down syndrome (Table 1). The proportion of children with Down syndrome born to young fathers (< 35 years) was 54% in California and 75% in the Czech Republic.

Paternal and maternal age of children with Down syndrome was, however, highly correlated: more in the Czech Republic (r = 0.75) than in California (r = 0.71) but both were highly significant (p < 0.001). Similar correlations were observed among non-Down syndrome children: correlation between paternal and maternal age was 0.74 in California and 0.72 in the Czech Republic. This strong correlation creates great potential for residual confounding of maternal age and the association between paternal age and Down syndrome.

In California, the highest proportion of infants was among mothers with secondary education (45.3% for all newborn babies and 42.3% for babies with Down syndrome; Table 1), whereas in the Czech Republic, it was among mothers with vocational education (41.9% for all newborn babies and 43.8% for babies with Down syndrome). The live birth prevalence of Down syndrome was the highest among mothers with the lowest education in California, and with the highest education level in the Czech Republic (Table 1). In California, the odds ratio of having child with Down syndrome was 1.99 (95% CI 1.52–2.62) for mothers with completed primary education compared to mothers with university education. In the Czech Republic, it was 0.84 (0.51–1.39).

Tables 2 and 3 show crude and adjusted odds ratios for live birth prevalence of Down syndrome by the sex of infants, education of mothers, maternal and paternal age for both study populations. After simultaneously controlling for 4 covariates, the effects of maternal education and maternal age increased in California, while in the Czech Republic there was no change in the effect of maternal education and a decrease in the effect of maternal age on the occurrence of Down syndrome. When additionally adjusted for paternal age (right panel in tables 2 and 3), the effect of maternal age is slightly reduced but remains highly significant. The effect of paternal age on live birth prevalence of Down syndrome is less clear. When adjusted for maternal age and education, the effect completely disappeared in California and was substantially reduced in the Czech Republic.

**Table 3:** Odds ratios (95% confidence interval) of occurrence of Down syndrome Czech Republic, 1994–98

	Crude	95% CI		Fully adjusted*	95% CI	
		Lower	Upper		Lower	Upper
*Sex of infants*						
Boy	1			1		
Girl	0.79	0.61	1.01	0.67	0.51	0.89

*Education of mother*						
Primary	0.84	0.51	1.39	1.03	0.57	1.86
Vocational	0.88	0.58	1.34	1.06	0.66	1.70
Secondary	0.76	0.49	1.17	0.85	0.53	1.36
University	1			1		

P for trend	*0.98*			*0.44*		

*Maternal age*						
-19	1			1		
20–22	1.31	0.76	2.25	0.89	0.46	1.69
23–25	1.12	0.64	1.93	0.67	0.33	1.33
26–28	1.28	0.72	2.25	0.76	0.37	1.60
29–31	1.44	0.79	2.62	0.61	0.27	1.41
32–34	2.56	1.39	4.69	1.31	0.57	3.03
35–37	2.68	1.32	5.45	1.39	0.55	3.56
38–40	2.96	1.23	7.13	1.00	0.28	3.52
41–43	8.99	3.54	22.82	4.61	1.38	15.40
44+	46.65	17.10	127.25	11.26	2.14	59.36
*Paternal age*						
-19	1			1		
20–22	0.63	0.18	2.19	0.68	0.19	2.41
23–25	1.09	0.34	3.51	1.33	0.40	4.50
26–28	1.03	0.32	3.32	1.34	0.39	4.63
29–31	0.94	0.29	3.08	1.23	0.35	4.40
32–34	1.24	0.37	4.12	1.49	0.41	5.46
35–37	1.95	0.58	6.53	1.98	0.52	7.44
38–40	2.21	0.63	7.69	2.02	0.51	7.96
41–43	2.10	0.54	8.14	1.68	0.38	7.42
44+	3.03	0.82	11.20	2.03	0.47	8.77

Note: * -Adjusted for all variables in the Table.

## Discussion

The main focus of the present study is to compare the epidemiological characteristics of Down syndrome in two contrasting populations, in California and the Czech Republic. Overall, prevalence of Down syndrome was significantly higher in Californian population. Most children with Down syndrome had parents younger than 35 years, however we found that the highest risk of having child with Down syndrome is among older parents (and particularly older mothers). We have also found that the risk of Down syndrome associated with increasing age increases more dramatically in California than in the Czech Republic. The association with paternal age was mostly explained when adjusted for maternal age. The association between maternal education and Down syndrome was much stronger in California than in the Czech Republic.

The effect of maternal age shown in this study is in agreement with several previous studies [7,20,21,23]. The removal of the effect of paternal age after adjustment for age of mother is very similar to recent analysis of Norwegian data [21] which showed relatively strong effect of paternal age almost completely explained by adjustment for maternal age. The association between paternal age and Down syndrome was also influenced by high proportion of missing information about age of fathers (about 8% in California and 17% in the Czech population). We looked at the association between education of mother and maternal age and proportion of missing data on paternal age. There was relationship between maternal education and missing paternal data: paternal data were available for 94% of children with university educated mother compared to only 52% available data for children with primary educated mothers. Paternal data were available for 87% of children with mothers aged 25–34 compared to 71% of children with mothers aged 40 and more, and 60% of children with mothers aged 19 or less.

Maternal age is, however, associated very strongly with Down syndrome. The association seems to be non-linear (much higher increase in prevalence of Down syndrome in older age than in younger ages). We tested for several polynomial functions but at the end we decided to use 3-years age groups as the model using age as categorical variable described the association between two variables the best.

The data on demographic and social characteristics are collected by the medical staff from medical records, identification cards, or self-reported by the new mothers. Although some misclassification of the independent variables could have occurred, it was probably small and random. The international comparison is based on data from two different sources. Thus, this study has the following potential limitation: the diagnosis of Down syndrome is slightly different in both populations. In California, Californian Birth Defect Monitoring program collects the information about congenital anomalies only for children up to the age of one year; in the Czech Republic this information is entered to the Register for all children up to 15 years of age. However, a high proportion of Down syndrome identification occurs in a very short period after birth; in the Czech Republic 95% of cases are diagnosed during the infant time period. It is not clear how this would bias our results.

The advanced health care systems – prenatal care in California and the Czech Republic provide a chance to compare the epidemiology of Down syndrome between these populations – the clinical practice is based on an approach that combines routine offer of maternal serum screening or amniocentesis to women with age 35 as a cut off for this procedure, or both. In the Czech Republic, prenatal diagnostics of Down syndrome (DS) is based on second trimester screening biochemical and ultrasound methods. An efficiency of DS prenatal diagnostics is around 66 – 67 % in the last years, that is, probably, a maximum for these methods. In order to increase the prenatal screning efficiency and to move the diagnostics towards earlier stages of pregnancy, methods of first trimester screening are gradually introduced. At present, first trimester screening is available at some prenatal dianostics departments and is expected to be implemented at other departments in early future. An increase of pre- and postnatally diagnosed DS cases at present is caused by several demographic and medical factors, such as by an increase of mean maternal age in the country along with increasing proportion of mothers 35 years of age old and over and increased number of multiple pregnancies. The current US prenatal testing guidelines recommend offering amniocentesis to women aged 35 years or older, or women who have been found by serum and ultrasound screening to be at a similarly high risk of giving birth to an infant with DS or another chromosal abnormality [24]. Although the prenatal testing for chromosomal disorders is of very high standards in both societies, other factors, such as the use of prenatal diagnostic services and accessibility of prenatal care may highly influence the prevalence levels. It is clear that abortion of affected fetuses play the important role in the occurrence of Down syndrome in the newborns. The selective use of prenatal diagnostic testing can have many implications in both comparative settings.

In the Czech Republic, very low live birth prevalence rate of Down syndrome (5.3 cases per 10,000 newborn infants) and a low proportion of children with Down syndrome born to women after 35-years of age (about 13%) supports consistent detection of this type of birth defect during pregnancy (almost 1 from 6 newborns had invasive diagnostic testing procedures during pregnancy, for example in 2003 14,984 from 93,185 newborns had diagnostic test) and a high ratio of terminated pregnancies. Congenital anomalies database (aggregated data by age of mother) for years 1994–1998 shows that only 42% of positively diagnosed Down syndromes were born. Among DS pregnancies of mothers 19 years old or younger, 55% children were born (and for age groups 20–22, 23–25 and 26–28 years, the proportions were 66%, 53% and 54%). However, only 13% were born to those aged 35 years or more [22] (+ Vladimir Gregor, personal communication). Rates of pregnancies with Down syndrome in 1994–98 are shown in table 4. No significant differences in prevalence of Down syndrome by maternal education in the Czech population are consistent with the fact that prenatal care is offered free and on the same qualitative level to all women.

**Table 4:** Prevalence rates of pregnancies with Down syndrome (per 10000), Czech Republic (1994–98)

**Age of mother**	**1994**	**1995**	**1996**	**1997**	**1998**	**Total**
-19	6.26	8.45	4.90	5.74	8.25	**6.70**
20–22	5.36	6.16	8.14	7.66	11.01	**7.36**
23–25	5.62	8.13	9.15	8.90	7.35	**7.80**
26–28	7.60	9.27	6.23	8.14	11.82	**8.69**
29–31	12.96	6.68	6.80	9.60	16.35	**10.57**
32–34	26.79	13.14	26.73	14.56	31.52	**22.66**
35–37	21.02	36.90	48.87	45.26	52.05	**40.93**
38–40	125.28	96.85	86.68	102	92.88	**101.07**
41–43	306.12	187.2	305.6	292.4	400	**297.03**
44+	333.33	615.4	169.5	204.1	317.5	**337.84**
						
**Total**	**10.94**	**11**	**12.34**	**12.32**	**16.29**	**12.50**

The educational gradient found in Californian sample might reflect selective impacts of maternity care, prenatal diagnosis, elective termination, and acceptance of prenatal diagnostic measures. We tried to test whether the association between education and Down syndrome can be confounded by ethnicity. When additional analysis including ethnicity of mother was conducted (results not presented), the association between maternal education and prevalence of Down syndrome was reduced but remained significant. Our assumption, that parents with lower socio-economic status often have few options for maternity care and little knowledge of prenatal testing is supported by the data from the birth certificates analysis (Table 5). The proportion of the amnio-utilization during pregnancy increased not only with maternal age but also with maternal education level. These results suggest that special support before and during the pregnancy would help to reduce social inequalities in prevalence of Down syndrome in California.

**Table 5:** Births with Amniocentesis by Mother's education and age, California, 1998 (in %)

Mother's education:		Mother's age:		
(in years)	- 19 years	20–34 years	35 + years	Total
0 – 8 years	0.3	0.4	2.0	0.6
9 – 11 years	0.3	0.5	4.0	0.6
12 – 15 years	0.5	0.9	11.1	2.2
16 + years	-	1.7	16.5	6.1
Total	0.4	0.9	11.5	2.5

Source: CDC, 1998, Birth Cohort Data Set

Previous results suggest that the rate of detection of Down syndrome may be higher in the Czech Republic than in the United States. The evaluation of the prenatal screening programs in Iowa and California showed that only approximately 40 to 50% of the cases were detected [25-27]. In the Czech Republic, 58% of pregnancies with DS were aborted in 1994–1998.

In California, the higher live birth prevalence rate of Down syndrome (11.5 cases per 10,000 newborn infants) and higher proportion of children with Down syndrome born to women older than 35 years (41%) might also reflect different attitudes towards prenatal diagnosis and abortion, different social and familial background and, maybe, a much more favorable opinion towards people with disabilities. Therefore it seems that the study results are strongly related to regional social context and abortion behavior.

As stated in the result section, the distribution of maternal age in two populations is different (more advanced maternal age in California). When birth prevalence rates in both countries are standardized by age of mother, difference in live birth prevalence of DS reduces (6.0 cases per 10,000 newborn infants in the Czech Republic and 10.0 in California) however it is still relatively large. It can be seen from table 1, that (with exception of 44+ age group), live birth prevalence of DS is higher in California in every age category.

At the beginning of the 1990s, the Czech Republic had one of the highest rates of therapeutic abortions in the world. The societal changes in the Czech Republic after 1989 have had a positive influence on the abortion situation. The abortion rate has significantly decreased and in 1994–1998 the induced abortion rate was 18.1 per 1,000 women of reproductive age (15–49). In the study period the number of newborns with Down syndrome was lower than the number of abortions with affected fetuses. A total of 251 newborns with Down syndrome and 396 electively aborted fetuses with Down syndrome were ascertained (data from Czech Congenital Anomalies Register); 61% of pregnancies with Down syndrome are electively aborted [22]. Although abortion incidence is the subject of research in the United States, nationally valid data are available from only two sources: the federal Centers for Disease Control and Prevention (CDC) and The Alan Guttmacher Institute. However the CDC does not collect abortion information from California specifically. In California, very limited statistics exist on abortion. The Alan Guttmacher Institute estimates the induced abortion rate was 31.2 per 1,000 women of reproductive age in California in 2000 [28]. Since December 2002 there have been restrictions on abortion in California – a woman must receive mandatory state-directed counseling before an abortion is provided. In the 1996–1997 California Genetic Disease Branch data set, a total of 456 positive pregnancies with Down syndrome were diagnosed via amniocenteses and 267 from them were electively aborted; the termination rate for these pregnancies was 58.5% (information obtained from the Department of Health Services, Genetic Disease Branch, Richmond, USA).

It is clearly recognized that Down syndrome prenatal screening is driven by several primary forces: (i) effort to reduce "the costs of life-long care" of people with Down syndrome through prenatal screening; (ii) clinical support for individual choices of mothers or couples; (iii) public health strategies designed to reduce birth defects and improving reproductive outcomes [14].

The social benefits of prenatal screening of Down syndrome are very important: (i) the prospective parents demand to be well-informed about their pregnancy outcomes, and (ii) the prospective parents need time to make informed decisions about selective prenatal termination of affected pregnancies or follow-up with it. When the pregnancy for Down syndrome affected child continues, the main goal is to support socially disadvantaged families, and to help to start lives of children born with Down syndrome.

## Conclusion

This study supports previous research showing that most children with Down syndrome are born to parents below 35 years of age and that significant risk levels for Down syndrome are not only in advanced maternal age categories. However risk of births with Down syndrome significantly increases with increasing paternal age, and, in particular, with increasing maternal age. Additionally, educational effects on maternal age-specific risk rates of Down syndrome were found for California mothers. The educational gradient might reflect selective impacts of maternity care, prenatal diagnosis, elective termination, and acceptance of prenatal diagnostic measures in the Californian population. On average, parents with lower socio-economic status often have few options for maternity care and little knowledge of prenatal testing, and they need special support for their start with parenthood and well-being of future generations. To prevent births of unwanted children with Down syndrome, comprehensive maternity care services must be available to all pregnant women regardless of socio-economic status. Prenatal diagnostic testing is also important for pregnant women at any age who would not consider abortion because babies with Down syndrome can need specialized care at delivery. Individuals with Down syndrome can live full, productive, and quality lives with help from modern medicine and lifetime educational/support programs. Access to the prenatal testing of chromosomal disorders to all pregnant women may be one possible task in strategy to reduce social inequalities in health.

## Competing interests

The author(s) declare that they have no competing interests.

## Authors' contributions

DD designed the study, collected the data, participated in data analyses and preparation of manuscript. HP analyzed the data and participated in preparation of manuscript. Both authors read and approved the final manuscript.

## Pre-publication history

The pre-publication history for this paper can be accessed here:


